# Leukotriene A4 Hydrolase Genotype and HIV Infection Influence Intracerebral Inflammation and Survival From Tuberculous Meningitis

**DOI:** 10.1093/infdis/jix050

**Published:** 2017-04-17

**Authors:** Nguyen T. T. Thuong, Dorothee Heemskerk, Trinh T. B. Tram, Le T. P. Thao, Lalita Ramakrishnan, Vu T. N. Ha, Nguyen D. Bang, Tran T. H. Chau, Nguyen H. Lan, Maxine Caws, Sarah J. Dunstan, Nguyen V. V. Chau, Marcel Wolbers, Nguyen T. H. Mai, Guy E. Thwaites

**Affiliations:** 1 Oxford University Clinical Research Unit,; 2 Pham Ngoc Thach Hospital for Tuberculosis and Lung Diseases, and; 3 Hospital for Tropical Diseases, Ho Chi Minh City, Vietnam;; 4 Nuffield Department of Medicine, University of Oxford,; 5 Department of Medicine, University of Cambridge, and; 6 Liverpool School of Tropical Medicine,United Kingdom; and; 7 Peter Doherty Institute for Infection and Immunity, University of Melbourne, Australia

**Keywords:** Leukotriene A4 hydrolase genotype, tuberculous meningitis, inflammatory response, cytokines, survival.

## Abstract

**Background.:**

Tuberculous meningitis (TBM) is the most devastating form of tuberculosis, yet very little is known about the pathophysiology. We hypothesized that the genotype of leukotriene A_4_ hydrolase (encoded by *LTA4H*), which determines inflammatory eicosanoid expression, influences intracerebral inflammation, and predicts survival from TBM.

**Methods.:**

We characterized the pretreatment clinical and intracerebral inflammatory phenotype and 9-month survival of 764 adults with TBM. All were genotyped for single-nucleotide polymorphism rs17525495, and inflammatory phenotype was defined by cerebrospinal fluid (CSF) leukocyte and cytokine concentrations.

**Results.:**

*LTA4H* genotype predicted survival of human immunodeficiency virus (HIV)–uninfected patients, with TT-genotype patients significantly more likely to survive TBM than CC-genotype patients, according to Cox regression analysis (univariate *P* = .040 and multivariable *P* = .037). HIV-uninfected, TT-genotype patients had high CSF proinflammatory cytokine concentrations, with intermediate and lower concentrations in those with CT and CC genotypes. Increased CSF cytokine concentrations correlated with more-severe disease, but patients with low CSF leukocytes and cytokine concentrations were more likely to die from TBM. HIV infection independently predicted death due to TBM (hazard ratio, 3.94; 95% confidence interval, 2.79–5.56) and was associated with globally increased CSF cytokine concentrations, independent of *LTA4H* genotype.

**Conclusions.:**

*LTA4H* genotype and HIV infection influence pretreatment inflammatory phenotype and survival from TBM. *LTA4H* genotype may predict adjunctive corticosteroid responsiveness in HIV-uninfected individuals.

**(See the editorial commentary by Fava and Schurr on pages 1011–3 and major article by Laarhoven et al on pages 1029–39.)**

Tuberculous meningitis (TBM) is the most severe form of disease caused by *Mycobacterium tuberculosis* [1]. It is characterized by a slowly progressive meningoencephalitis with necrotizing, granulomatous inflammation predominantly affecting the basal meninges. Inflammatory exudates can obstruct the passage of cerebrospinal fluid (CSF), leading to hydrocephalus; small and medium-sized intracerebral arteries can become inflamed and occluded, leading to infarcts; and granulomas may enlarge to form tuberculomas, which can cause mass effects and seizures [1, 2]. Death or neurological disability occurs in around 50% of cases.

There is a long-standing hypothesis that death from TBM results from an excessive intracerebral inflammatory response [3]. The corollary of this hypothesis has been that adjunctive antiinflammatory treatment with corticosteroids (eg, dexamethasone) improves survival, which has been demonstrated in predominantly human immunodeficiency virus (HIV)–uninfected individuals in a small number of trials [4]. Yet how corticosteroids improve survival and whether they do so in HIV-infected patients remain uncertain [5–7].

We recently identified a common functional promoter variant (rs17525495; C/T transition 12 bp upstream of the transcription start site) in the gene encoding leukotriene A_4_ hydrolase (*LTA4H*) that appeared to predict survival and dexamethasone responsiveness in HIV-uninfected adults with TBM [8]. This human candidate gene association study was guided by findings in a zebra fish model where LTA4H was found to determine the balance of proinflammatory and antiinflammatory eicosanoids in response to mycobacterial infection [8, 9]. In humans, *LTA4H* rs17525495 allele homozygosity (TT and CC) was associated with susceptibility to mycobacterial infection, but the associations involved opposing inflammatory states—high inflammation for the TT genotype and low inflammation for the CC genotype [8]. In comparison, heterozygotes (CT) had an intermediate inflammatory response and were more likely to survive TBM [8]. In a retrospective study analyzing HIV-uninfected adults with TBM enrolled into a randomized controlled trial of adjunctive dexamethasone [6], we found that the survival benefit of dexamethasone was restricted to patients with the hyperinflammatory *LTA4H* genotype, TT, with possible harm suggested in patients with the hypoinflammatory genotype, CC [8]. These preliminary findings suggested that *LTA4H* genotype might be a critical determinant of inflammation and consequently of the response to adjunctive antiinflammatory treatment.

To investigate these possibilities further and the influence of HIV infection on inflammation and survival, we designed a prospective study, using a new cohort of 764 Vietnamese adults with TBM enrolled into a randomized controlled trial of intensified antituberculosis therapy, the results of which are reported elsewhere [10]. All of the patients received adjunctive dexamethasone. We have used these carefully characterized patients to address the following hypotheses: (1) *LTA4H* genotype predicts survival of patients with TBM receiving corticosteroids, (2) *LTA4H* genotype determines pretreatment CSF inflammatory phenotype, (3) death from TBM is associated with an inadequate intracerebral inflammatory response, and (4) HIV infection is associated with an attenuated CSF inflammatory response [11].

## METHODS

### Participants

As previously described [10], 817 Vietnamese adults (age, >17 years) were enrolled into a randomized controlled trial of intensified antituberculosis chemotherapy between April 2011 and June 2014 and were followed for 9 months. Peripheral blood and cerebrospinal fluid (CSF) specimens were collected on enrollment and stored at −80ºC for later analysis. Fifty-three patients were excluded from the current study: 22 did not have TBM, 5 did not consent to genetic testing, 9 died early or left the study before the blood specimen was collected, and 17 had either insufficient DNA concentrations or experienced failure of genotyping analysis.

Written informed consent was obtained from each patient or from an accompanying relative if the patient could not provide consent. Protocols were approved by the Oxford Tropical Research Ethics Committee in the United Kingdom, the institutional review boards of the Hospital for Tropical Diseases and Pham Ngoc Thach Hospital for Tuberculosis and Lung Disease, and the Ethical Committee of the Ministry of Health in Vietnam.

### Treatment

All patients were randomly allocated to treatment with each standard antituberculosis regimen consisting of isoniazid (5 mg/kg/day; maximum, 300 mg/day), rifampicin (10 mg/kg/day), pyrazinamide (25 mg/kg/day; maximum, 2 g/day), and ethambutol (20 mg/kg/day; maximum, 1.2 g/day) for 3 months, followed by rifampicin and isoniazid at the same doses for a further 6 months, or with an intensified regimen that consisted of the standard regimen with an additional higher dose of rifampicin (15 mg/kg/day) and levofloxacin (20 mg/kg/day) for the first 8 weeks of treatment. All patients received adjunctive dexamethasone for the first 6–8 weeks of treatment [6], [12]. In HIV-infected patients, antiretroviral therapy (ART) started prior to enrollment was continued unless there were contraindications with rifampicin [10]. For ART-naive patients, ART was started after 8 weeks of antituberculosis therapy [13].

### Clinical and CSF Inflammatory Phenotyping

Detailed clinical assessments were made at baseline and up to 9 months after randomization. Baseline disease severity was assessed by the modified British Medical Research Council (BMRC) grading system [14]. An HIV test was performed on all patients at enrollment. Concentrations of total and differential blood cell counts (including CD4^+^ T-cell counts in HIV-infected patients) and CSF leukocyte counts, lactate levels, glucose levels, and protein levels were measured by standard methods.

A panel of 10 human cytokines was measured in stored CSF specimens by Luminex multiplex bead array technology (Bio-Rad Laboratories, Hercules, CA). The panel consists of interleukin 1β (IL-1β), IL-2, IL-4, IL-5, IL-6, IL-10, IL-12p70, IL-13, interferon γ (IFN-γ), and tumor necrosis factor α (TNF-α). Assays were read on the Bio-Plex 200 platform, and the Bio-Plex Manager software 6.0 was used for bead acquisition and analysis. Additional details on the method for cytokine measurement is provided in the Supplementary Materials.

### LTA4H Genotyping

*LTA4H* rs17525495 (C/T) polymorphism was genotyped by TaqMan genotyping assay [15] and confirmed by sequencing. For Taqman genotyping, the assay mixture (LightCycler 480 Probes Master, Roche) was preloaded with 1 pair of primers, to amplify the polymorphic sequence, and 2 fluorescently labeled allele-specific probes (TaqMan predesigned SNP genotyping assays, Applied Biosystems), and the fluorescent signals were captured by real-time polymerase chain reaction analysis (Light Cycler 480, Roche). For sequencing, the primers 5’-TTCACCCATCCCCCAAC-3’ and 5’-GGG TGCTGTGAGAGATCTG-3’ were used with the Bigdye Terminator v3.1 Cycle Sequence kit (Applied BioSystems) on the Genetic Analyzer machine (Applied BioSystems).

### Statistical Analysis

Cox regression was used to model the univariable and joint effects of predefined risk factors, including *LTA4H* genotype and antituberculosis treatment regimen allocation, on 9-month survival. Kaplan–Meier estimates were used to visualize survival functions. Comparisons of cytokine concentrations between HIV-infected and HIV-uninfected patients were based on the Wilcoxon rank sum test, comparisons of cytokines by disease severity or *LTA4H* genotype were based on a linear trend test implemented using robust linear regression, and the effect of log-transformed cytokines on survival was assessed via univariable Cox regression. For each cytokine analysis, we performed resampling-based multiple hypothesis testing to correct for multiplicity [16]. Statistical analyses were performed using the statistical package R v3.0.1 [17].

## RESULTS

### Characteristics of the Population

The baseline clinical characteristics of the 764 adults included in this study are given in Supplementary Table 1. The majority (630 [82.5%]) had BMRC grade 1 or 2 disease severity at enrollment, 325 (42.5%) were infected with HIV, and 395 (51.8%) had microbiologically confirmed disease. HIV-infected patients were significantly more likely than HIV-uninfected patients to be male, to be younger, to weigh less, to have previously received tuberculosis treatment, and to have microbiologically confirmed TBM. The 9-month case-fatality rate was 27.7% (212 of 764) overall, 18.9% (83 of 439) among patients without HIV infection, and 39.7% (129 of 325) among those with HIV infection (Supplementary Figure 1). Eighty-nine patients (11.6%) had the TT genotype at *LTA4H* rs17525495, 345 (45.2%) had CT, and 330 (43.2%) had CC, and this polymorphism was in Hardy-Weinberg equilibrium (*P* = .995).

### Hypothesis 1: *LTA4H* Genotype Predicts Survival of Patients With TBM Receiving Corticosteroids

**Figure 1.**
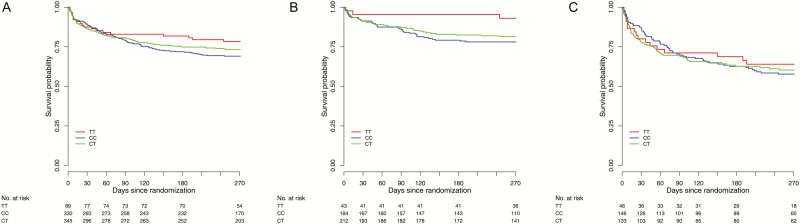
Kaplan–Meier survival curves stratified by *LTA4H* genotype. Survival in all patients with tuberculous meningitis (*A*), those without human immunodeficiency virus (HIV) infection (*B*), and those with HIV infection (*C*). In HIV-uninfected patients, case-fatality rates were 7.1% (3 of 42) in those with genotype TT, 21.4% (40 of 187) in those with genotype CT, and 18.7% (39 of 209) in those with genotype CC. In HIV-infected patients, case-fatality rates were 34.8% (16 of 46) in those with genotype TT, 42.1% (61 of 145) in those with genotype CT, and 38.8% (52 of 134) in those with genotype CC. Overall likelihood ratio test for an effect of *LTA4H* genotype on survival revealed *P* values of .24 for all patients, .05 for HIV-uninfected patients (*P* = .08 for the effect of TT vs CT, and *P* = .04 for the effect of TT vs CC), and .87 for HIV-infected patients.

Our previous findings suggested that dexamethasone increased survival among HIV-uninfected adults with TBM who had the *LTA4H* TT genotype but had uncertain and possibly harmful effects in those who had the CT and CC genotypes [8]. In this study, intensified antituberculosis chemotherapy was not associated with improved survival in all patients, as previously reported [10], or in patients stratified by *LTA4H* genotype (Supplementary Figure 1). Univariable analysis indicated that *LTA4H* genotype had no significant impact on survival in all patients, but subdivision by HIV status found increased survival in HIV-uninfected patients with the TT genotype, compared with patients with the other 2 genotypes, as predicted (overall likelihood ratio test *P* = .05; Figure 1 and Table 1). The survival rate among CT heterozygotes was more similar to that for CC homozygotes than for TT homozygotes. Adjustment for predefined risk factors, including age, weight, BMRC grade, intensified regimen, drug resistance, and CSF leukocyte numbers, did not substantially alter the size or strength of the *LTA4H* genotype association with survival in the HIV-uninfected patients (overall likelihood ratio test *P* = .03; Table 1).

**Table 1.** Univariable Effect of *LTA4H* on the Hazard of Death and Cox Regression–Based Multivariable Effect of Other Risk Factors on the Hazard of Death, Overall and Stratified by Human Immunodeficiency Virus (HIV) Status

Variable	Overall, HR (95% CI) (n = 764)^a^	*P*	HIV Uninfected, HR (95% CI) (n = 439)^a^	*P*	HIV Infected, HR (95% CI) (n = 325)^a^	*P*
*LTA4H* genotype (univariable effect)^b^						
TT	1 (reference)		1 (reference)		1 (reference)	
CT	1.26 (.77–2.07)	.35	2.83 (.88–9.17)	.08	1.14 (.65–2.00)	.65
CC	1.47 (.90–2.39)	.12	3.40 (1.05–11.00)	.04	1.16 (.67–2.01)	.60
**Multivariable Cox regression model**						
*LTA4H* genotype (adjusted effect)^c^						
TT	1 (reference)		1 (reference)		1 (reference)	
CT	1.62 (.95–2.75)	.07	2.47 (.76–8.05)	.13	1.54 (.78–3.04)	.21
CC	1.65 (.98–2.79)	.06	3.51 (1.08–11.43)	.037	1.47 (.76–2.85)	.25
Age (per 10-y increase)	1.34 (1.20–1.50)	<.0001	1.45 (1.28–1.66)	<.0001	1.01 (.73–1.41)	.94
Weight (per 10-kg increase)	0.77 (.64–.92)	.005	0.91 (.69–1.18)	.47	0.61 (.46–.81)	.0007
HIV infected	3.94 (2.79–5.56)	<.0001	…		…	
BMRC grade^d^						
1	1 (reference)		1 (reference)		1 (reference)	
2	2.09 (1.44–3.04)	.0001	2.71 (1.38–5.3)	.004	1.52 (.92–2.54)	.11
3	6.71 (4.54–9.93)	<.0001	7.25 (3.52–14.94)	<.0001	5.22 (3.13–8.70)	<.0001
Hyperintensive regimen	0.94 (.71–1.25)	.69	1.06 (.68–1.68)	.79	1.15 (.77–1.72)	.50
ART at enrollment	…		…		0.65 (.41–1.03)	.07
Resistance category						
No INH or RIF resistance	1 (reference)		1 (reference)		1 (reference)	
INH resistance^e^	0.99 (.61–1.61)	.98	0.55 (.21–1.44)	.22	1.00 (.53–1.88)	1.00
RIF/multidrug resistance^f^	3.70 (1.84–7.46)	.0003	1.90 (.24–14.78)	.54	5.78 (2.58–12.92)	<.0001
Unknown resistance^g^	1.30 (.91–1.85)	.15	0.80 (.43–1.47)	.47	1.75 (1.06–2.89)	.30
CSF leukocyte count (per 2-fold increase)	0.88 (.83–.93)	<.0001	0.85 (.77–.94)	.001	0.95 (.87–1.03)	.18
Baseline CD4^+^ T-cell count (per 100- cells/mm^3^ increase)	…		…		0.59 (.40–.87)	.007

Abbreviations: ART, antiretroviral therapy; CI, confidence interval; CSF, cerebrospinal fluid; HR, hazard ratio; INH, isoniazid; RIF, rifampicin.

^a^Patients with missing covariates were excluded from the multivariable analysis (26 in the overall group, 14 in the HIV-uninfected group, and 55 in the HIV-infected group, mainly due to missing CD4^+^ T-cell counts).

^b^Overall likelihood ratio test for significance of *LTA4H*: *P* = .24 (overall), *P* = .05 (HIV uninfected), and *P* = .87 (HIV infected).

^c^Overall likelihood ratio test for significance of *LTA4H*: *P* = .12 (overall), *P* = .03 (HIV uninfected), and *P* = .41 (HIV infected). Variables used for adjustment in multivariable Cox regression are as listed in the first column.

^d^Modified British Medical Research Council (BMRC) disease severity criteria are as follows: grade 1, Glasgow coma scale (GCS) score of 15, with no neurologic signs (baseline); grade 2, GCS score of 11–14 (or 15, with focal neurologic signs); and grade 3, a GCS score of ≤10.

^e^Resistance to INH, with or without other resistance, but no RIF resistance.

^f^Multidrug resistance to at least rifampicin RIF and INH, or isolated RIF resistance from culture results.

^g^Patients for whom no drug resistance results were available.

In HIV-infected patients, the effect of *LTA4H* genotype on survival was less pronounced and did not reach statistical significance (Figure 1 and Table 1). The evidence that HIV-status modified the effect of *LTA4H* genotype on survival was only weak (*P* for interaction = .168). Table 1 also presents findings of multivariable Cox regression analysis of other predefined risk factors associated with death from TBM, confirming the prognostic importance of age, weight, HIV infection, BMRC grade, drug resistance, and CSF leukocyte numbers.

These findings support the hypothesis that *LTA4H* genotype is associated with survival, independently of other risk factors, in HIV-uninfected patients with TBM.

### Hypothesis 2: *LTA4H* Genotype Predicts Inflammatory Phenotype

We surmised that because LTA4H directly affects leukotriene B_4_, a potent neutrophil and macrophage chemoattractant, *LTA4H* genotype might influence the inflammatory phenotype. To test this hypothesis, we compared pretreatment CSF leukocyte and cytokine concentrations across *LTA4H* genotypes in both HIV-infected and HIV-uninfected patients. While the TT genotype was associated with increased CSF leukocyte counts in a previous cohort [8], this association was not found in this cohort in either HIV-uninfected patients or HIV-infected patients (Supplementary Tables 2–4).

**Figure 2.**
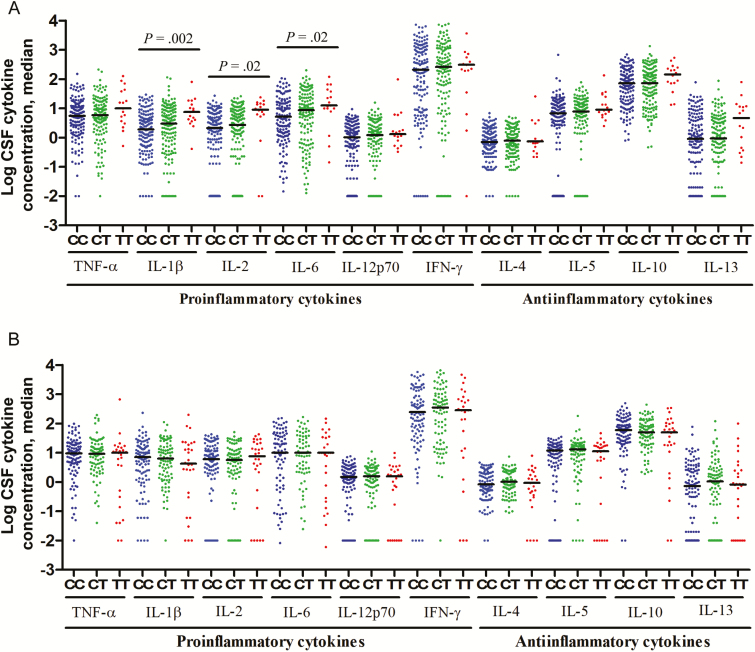
Cerebrospinal fluid (CSF) levels of cytokine expression, by *LTA4H* genotype, in human immunodeficiency virus (HIV)–uninfected (*A*) and HIV-infected (*B*) patients with tuberculous meningitis. Concentrations are in picograms/milliliter for all cytokines except interleukin 6 (IL-6), for which concentrations are in nanograms/milliliter. *A*, Data are for 147 patients with *LTA4H* genotype CC, 141 with genotype CT, and 16 with genotype TT. *B*, Data are for 104 patients with *LTA4H* genotype CC, 87 with genotype CT, and 28 with genotype TT. Statistical comparisons were made using a linear trend test corrected for multiple testing across the 10 cytokines. Only *P* values of ≤ .05 are shown. Abbreviations: IFN-γ, interferon γ; TNF-α, tumor necrosis factor α.

As a more comprehensive readout of inflammation in the CSF, we examined cytokine concentrations. In HIV-uninfected patients, there was a significant association between *LTA4H* genotype and CSF IL-1β, IL-2, and IL-6 expression, with low concentrations of each cytokine in patients with genotype CC, intermediate concentrations in those with genotype CT, and high concentrations in those with genotype TT (Figure 2A). In the zebra fish, LTA4H levels mediate their effects on pathogenesis through modulating TNF-α levels [8]. In our cohort, CSF TNF-α concentrations had a similar trend as those of the other cytokines, but the comparison across *LTA4H* genotype did not reach statistical significance (raw *P* = .14; multiplicity-adjusted *P* = .49). This may be because dampened signal-to-noise levels due to low baseline levels of this more upstream cytokine regulated downstream signaling pathways, causing amplification of pathways further downstream [18].

Higher expression of CSF cytokines (ie, IL-1β, IL-2, and IL-6) and increased global cytokine expression support the hypothesis that *LTA4H* genotype influences pretreatment CSF inflammatory phenotype, but only for those uninfected with HIV. This effect appears to be independent of disease severity, which was similar across the genotypes, along with other baseline clinical characteristics (Supplementary Tables 2–4). Unlike uninfected patients, *LTA4H* genotype did not affect CSF cytokine concentrations in HIV-infected patients (Figure 2B).

### Hypothesis 3: Hypoinflammation Is Associated With Death

Given the long-standing hypothesis that excessive intracerebral inflammation is strongly linked to death from TBM, we next sought to investigate the relationship between pretreatment inflammatory CSF phenotype and the risk of death. In this trial, all patients received dexamethasone. Taking into account the observation from the previous trial that dexamethasone is protective in those with a predisposition for a hyperinflammatory response, it is conceivable that, with exposure to corticosteroids, patients with an inadequate inflammatory response will have worse outcome. In all patients, univariable analysis demonstrated that lower CSF leukocyte counts were significantly associated with death (*P* < .0001), with median values of 59 × 10^3^ cells/mL (interquartile range [IQR], 13–240 × 10^3^ cells/mL) in those who died and 135 × 10^3^ cells/mL (IQR, 48–298 × 10^3^ cells/mL) in survivors. The association was similar in HIV-uninfected patients and HIV-infected patients: in HIV-uninfected patients, median values in those who died were 47 × 10^3^ cells/mL (IQR, 12–212 × 10^3^ cells/mL), compared with 124 × 10^3^ cells/mL (IQR, 48–278 × 10^3^ cells/mL) in survivors (*P* = .002); in HIV-infected patients, median values in those who died were 77 × 10^3^ cells/mL (IQR, 15–270 × 10^3^ cells/mL), compared with 153 × 10^3^ cells/mL (IQR, 46–382 × 10^3^ cells/mL) in survivors (*P* < .0001). In the multivariable analysis, the association only retained significance in HIV-uninfected patients (*P* = .001; Table 1).

**Figure 3.**
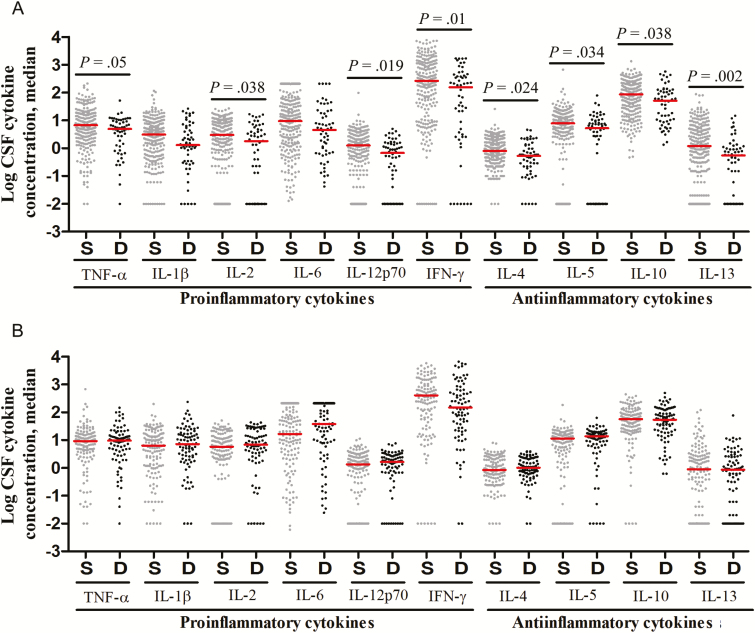
Cerebrospinal fluid (CSF) levels of cytokine expression, by patients outcome, in human immunodeficiency virus (HIV)–uninfected (*A*) and HIV-infected (*B*) patients with tuberculous meningitis. Concentrations are in picograms/milliliter for all cytokines except interleukin 6 (IL-6), for which concentrations are in nanograms/milliliter. *A*, Data are for 248 patients with an outcome of survival (S) and 56 with an outcome of death (D). *B*, Data are for 133 patients with an outcome of S and 86 with an outcome of D. Statistical comparisons were based on Cox regression models of the univariable effect of (log-transformed) CSF cytokines expression levels on 9-month survival corrected for multiple testing across the 10 cytokines. Only *P* values of ≤ .05 are shown. Abbreviations: IFN-γ, interferon γ; TNF-α, tumor necrosis factor α.

Figure 3 presents pretreatment CSF cytokine concentrations in those who survived or died, with subgroups defined by HIV infection. In HIV-uninfected patients, there was a striking association between death and lower cytokine concentrations (Figure 3A). In HIV-infected patients, however, cytokine concentrations were not different between the 2 groups (Figure 3B).

These data support the notion that poor outcome from TBM, in the context of immunosuppressive treatment, is associated with an inadequate pretreatment inflammatory response in HIV-uninfected individuals. Taken together with the associations between *LTA4H* genotype and both inflammatory phenotype and survival, these findings suggest that patients with the CC genotype in particular are dying because of an inadequate inflammatory response and that adjunctive dexamethasone may be harmful to these patients.

**Figure 4.**
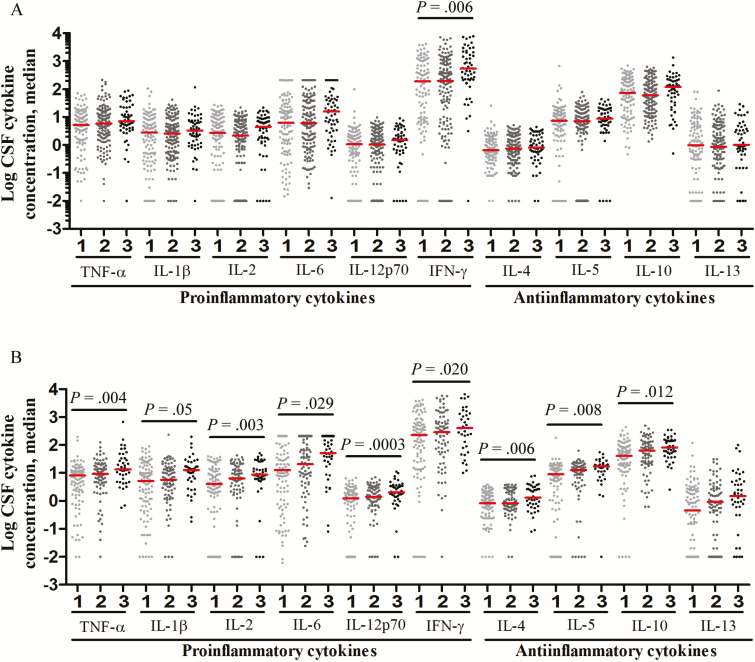
Cerebrospinal fluid (CSF) levels of cytokine expression, by modified British Medical Research Council (BMRC) grade 1, grade 2, and grade 3 disease severity, in human immunodeficiency virus (HIV)–uninfected (*A*) and HIV-infected (*B*) patients with tuberculous meningitis. Concentrations are in picograms/milliliter for all cytokines except interleukin 6 (IL-6), for which concentrations are in nanograms/milliliter. *A*, Data are for 108 patients with BMRC grade 1 disease severity, 141 with grade 2, and 55 with grade 3. *B*, Data are for 98 patients with BMRC grade 1 disease severity, 79 with grade 2, and 42 with grade 3. Statistical comparisons were made using a linear trend test corrected for multiple testing across the 10 cytokines. Only *P* values of ≤ .05 are shown. Abbreviations: IFN-γ, interferon γ; TNF-α, tumor necrosis factor α.

These associations could be confounded by disease severity at the start of treatment (BMRC grade), a major independent determinant of survival from TBM (Table 1). Disease severity was not associated with significant differences in CSF leukocyte numbers or types (data not shown). However, elevated CSF cytokine concentrations were associated with more-severe BMRC grades (Figure 4), especially in HIV-infected patients, in whom increasing BMRC grade was significantly associated with increased concentrations for all measured cytokines (Figure 4B). Higher BMRC grade strongly predicted death from TBM (Table 1) and was closely linked to more-severe intracerebral inflammation (Figure 4). Yet death was not associated with increased CSF cytokine concentrations (Figure 3). Indeed, in HIV-uninfected individuals, death was associated with significantly decreased CSF cytokine concentrations (Figure 3A). This finding supports the idea that another variable, possibly *LTA4H* genotype, influences the relationship between intracerebral inflammation, disease severity, and death.

### Hypothesis Four: HIV Infection Is Associated With an Attenuated CSF Inflammatory Response

**Figure 5.**
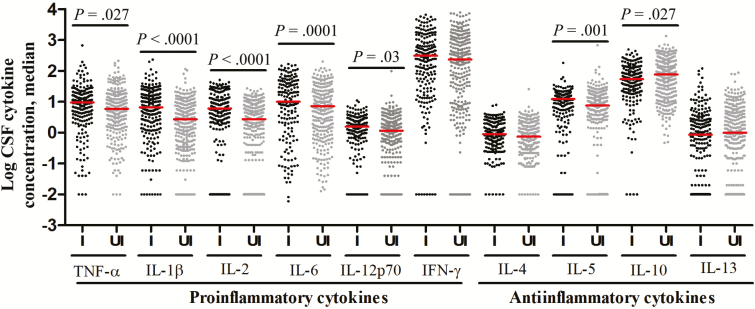
Cerebrospinal fluid (CSF) levels of cytokine expression in human immunodeficiency virus (HIV)–infected (I) and HIV-uninfected (UI) patients with tuberculous meningitis. Concentrations are in picograms/milliliter for all cytokines except interleukin 6 (IL-6), for which concentrations are in nanograms/milliliter. Statistical comparisons between HIV-infected and HIV-uninfected were made by the Mann–Whitney test corrected for multiple testing across the 10 cytokines. Only *P* values of ≤ .05 are shown. Abbreviations: IFN-γ, interferon γ; TNF-α, tumor necrosis factor α.

HIV infection is a strong independent predictor of death from TBM (Table 1), which may be due to an inadequate immune response. We compared the pretreatment CSF cells and cytokine profiles of all HIV-infected and HIV-uninfected patients. Intracerebral leukocytes counts were not different, while mean neutrophil percentages were higher in HIV-infected patients than in HIV-uninfected patients (17% vs 5%; *P* < .0001; Supplementary Table 1). HIV infection was associated with an unexpected global increase in cytokine expression (Figure 5), contradicting our hypothesis. Only the antiinflammatory cytokine IL-10, which inhibits the immune response to *M. tuberculosis* [19], was significantly lower (*P* = .027) in HIV-infected patients, and the CSF ratio of INF-γ to IL-10, an index of proinflammatory versus antiinflammatory cytokines, showed a significant excess of INF-γ in HIV-infected patients (*P* < .0001). Pearson correlations between the absolute neutrophil count and the 10 log-transformed CSF cytokine concentrations were all positive, ranging from 0.08 to 0.47, and all concentrations were significantly different from 0 (raw *P* < .05), except for the IL-13 concentration (*P* = .09).

The degree of immune suppression may alter these relationships and in turn the influence of *LTA4H* on the inflammatory response. CSF cytokine expression profiles were not different in those with prior ART (data not shown) but were influenced by CD4^+^ T-cell count. We arbitrarily categorized patients on the basis of CD4^+^ T-cell counts of ≥150 or <150 cells/µL and compared CSF inflammation between both groups and patients without HIV infection. We found that patients with the highest level of immunosuppression (ie, those with a CD4^+^ T-cell count of <150) had a significantly higher median CSF neutrophil percentage (25%) than patients with a CD4^+^ T-cell count of ≥150 cells/µL (10%; *P* = .021) and patients without HIV infection (5%; *P* < .0001); patients with the highest level of immunosuppression also had significantly greater cytokine concentrations than patients in the other 2 groups (Supplementary Figure 2*A*). Indeed, the patients with a CD4^+^ T-cell count of ≥150 cells/μL had a similar CSF cytokine profile to HIV-uninfected patients, with no significant difference in concentrations for any cytokine in the multivariable analysis.

Forty-four percent of patients (105 of 238) with a CD4^+^ T-cell count of <150 cells/μL died, compared with 13% (5 of 39) with a CD4^+^ T-cell count of ≥150 cells/µL and 19% (83 of 439) without HIV infection. *LTA4H* did not appear to influence survival in patients with a CD4^+^ T-cell count of <150 cells/µL (Supplementary Figure 2*B*), but in those with a CD4^+^ T-cell count of ≥150 cells/µL (Supplementary Figure 2*C*), the stratified plot by *LTA4H* genotype was visually similar to the corresponding plot for HIV-uninfected patients (Figure 1). This analysis was underpowered and exploratory, but these data suggest *LTA4H* may influence intracerebral inflammation and dexamethasone-associated survival in HIV-infected patients with higher CD4^+^ T-cell counts and warrants further investigation.

## DISCUSSION

Our previous work suggested that *LTA4H* genotype may be central to the inflammatory response in TBM and might predict who benefits from adjunctive corticosteroid treatment [8]. Here, we extended these original observations in a new cohort of 764 prospectively characterized Vietnamese adults with TBM, all of whom received corticosteroids.

First, we found that *LTA4H* genotype influences the survival of HIV-uninfected patients with TBM treated with dexamethasone (Figure 1 and Table 1). Patients with the TT genotype were significantly more likely to survive TBM than those with the CC genotype, based on both univariable and multivariable analyses (Table 1), which confirms our previous findings. Furthermore, we found that patients with the *LTA4H* TT genotype had high CSF proinflammatory cytokine concentrations (IL-1β, IL-2, and IL-6), with intermediate and lower concentrations in those with the CT and CC genotypes, respectively (Figure 2). This suggests that the suppression of inflammation by dexamethasone confers a survival benefit on patients with the TT genotype but may be indifferent or even harmful in the other 2 genotype groups.

Second, to test the model in which the best outcomes are associated with an intermediate inflammatory response, as opposed to either high or low responses, we investigated the relationships between disease severity, CSF inflammatory phenotype, HIV infection, and survival from TBM. We found that increased pretreatment CSF cytokine concentrations correlated with more-severe disease. In addition, we observed that, in HIV-uninfected patients, death was strongly associated with an attenuated inflammatory response. Patients with low pretreatment CSF leukocyte and cytokine concentrations were more likely to die from TBM. This paradoxical finding may be explained by an *LTA4H*-dependent effect of dexamethasone upon survival: patients with the hyperinflammatory TT genotype benefit from immune suppression, whereas those with the hypoinflammatory CC genotype do not.

HIV infection is well described as an independent predictor of death from TBM, but its impact on pathogenesis is poorly understood. Surprisingly, death was not associated with high or low cytokine expression, but neutrophil and cytokine concentrations overall were significantly higher in HIV-infected patients than in uninfected patients, with the exception of IL-10. Our data confirm previous findings that CSF ratios of INF-γ to IL-10 are inclined toward excess INF-γ in HIV-positive patients [11] and support the possibility that the alteration skews the balance towards a T-helper type 1 response. Moreover, positive correlations between CSF neutrophil numbers and cytokine concentrations suggest a significant role for neutrophils in the immunopathogenesis. In the early stage of disease in HIV-infected individuals, neutrophils may accumulate at infected sites, and overproduction of cytokines (particularly TNF-α) may be responsible for tissue damage and contribute to pathology [20–23].

*LTA4H* genotype did not have a significant influence upon pretreatment inflammatory phenotype and survival in HIV-infected patients. Instead, HIV appears to drive a dysregulated hyperinflammatory phenotype with very poor outcomes. We hypothesized, however, that these effects may be mitigated by higher CD4^+^ T-cell counts. Indeed, we found that patients with CD4^+^ T-cell counts of ≥150 cells/μL had a CSF inflammatory phenotype and survival rate similar to those for HIV-uninfected patients and showed a similar albeit nonsignificant link between *LTA4H* genotype and survival.

Previous studies in zebra fish showed that LTA4H regulates the balance between proinflammatory and antiinflammatory eicosanoids, namely leukotriene B_4_ and lipoxin A_4_ [8, 9]. To study the association of *LTA4H* genotype and concentrations of these 2 metabolites, we measured the metabolites in CSF samples from patients with TBM, using enzyme-linked immunosorbent assay. Leukotriene B_4_ levels were undetectable in around 50% of samples, and lipoxin A_4_ assays behaved differently on 2 occasions of measurement (data not shown). Therefore, no conclusion could be drawn on the regulation of leukotriene B_4_ and lipoxin A_4_ by *LTA4H* in the CSF of patients with TBM in this study.

In conclusion, these new data suggest that *LTA4H* and HIV influence both pretreatment CSF inflammatory phenotype and survival from TBM. We propose a new model for TBM pathogenesis whereby both attenuated and excessive inflammatory responses are linked to poor survival. This model could have profound implications for the use of adjunctive antiinflammatory corticosteroids, currently recommended for all patients with TBM [24]. Substantial further work is required, however, to validate the model and to determine whether current therapeutic approaches need revision. In particular, our findings need validation in non-Vietnamese populations, the mechanism by which *LTA4H* alters CSF cytokine expression and treatment response in patients with TBM needs elucidation, and whether HIV status and *LTA4H* genotype should be used to select patients for adjunctive corticosteroid needs to be addressed in randomized controlled trials.

## Supplementary Data

Supplementary materials are available at *The Journal of Infectious Diseases* online. Consisting of data provided by the authors to benefit the reader, the posted materials are not copyedited and are the sole responsibility of the authors, so questions or comments should be addressed to the corresponding author.

## Supplementary Material

Supplementary Figure S1

Supplementary Figure S2

Supplementary Data

## Notes

***Acknowledgments.*** We thank the participants in the study and the physicians and nurses at HTD and PNT hospitals who were responsible for their care and follow-up; Tran Dinh Dinh, for her help in confirming *LTA4H* genotyping by sequencing; and David Tobin and Laura Whitworth, for their helpful comments on the manuscript.

***Financial support.*** This work was supported by the Wellcome Trust (097124/Z/11/Z to N. T. T. T.) and the Li Ka Shing Foundation (to D. H.).

***Potential conflicts of interest.*** All authors: No reported conflicts. All authors have submitted the ICMJE Form for Disclosure of Potential Conflicts of Interest. Conflicts that the editors consider relevant to the content of the manuscript have been disclosed.
