# Supplementary figures and images for: Leukotriene A4 Hydrolase Genotype and HIV Infection Influence Intracerebral Inflammation and Survival From Tuberculous Meningitis
Source: J Infect Dis. 2017 Apr 17;215(7):1020–8. doi: 10.1093/infdis/jix050 (PMC5426373; doi:10.1093/infdis/jix050)

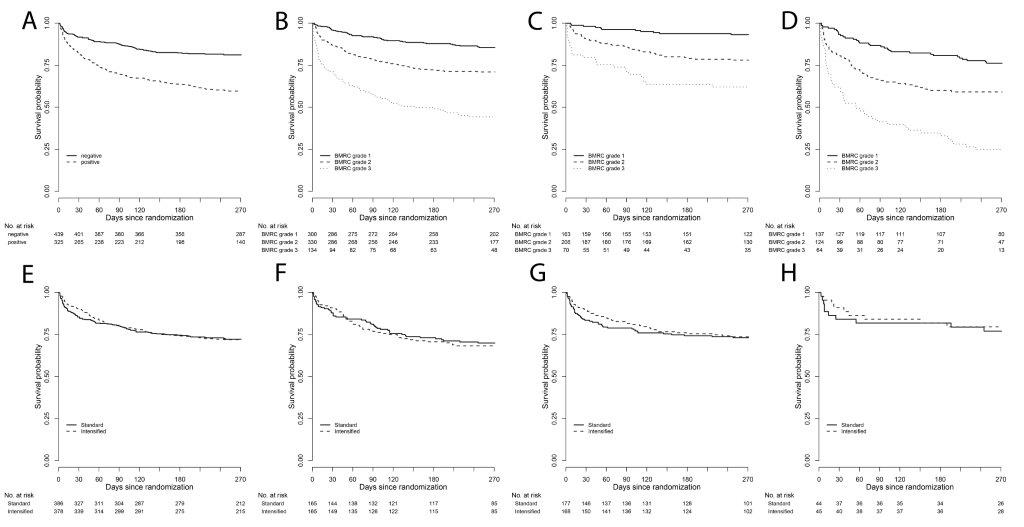

Supplement: Supplementary Figure S1 [file jix050_suppl_Supplementary_Figure_S1.jpeg]

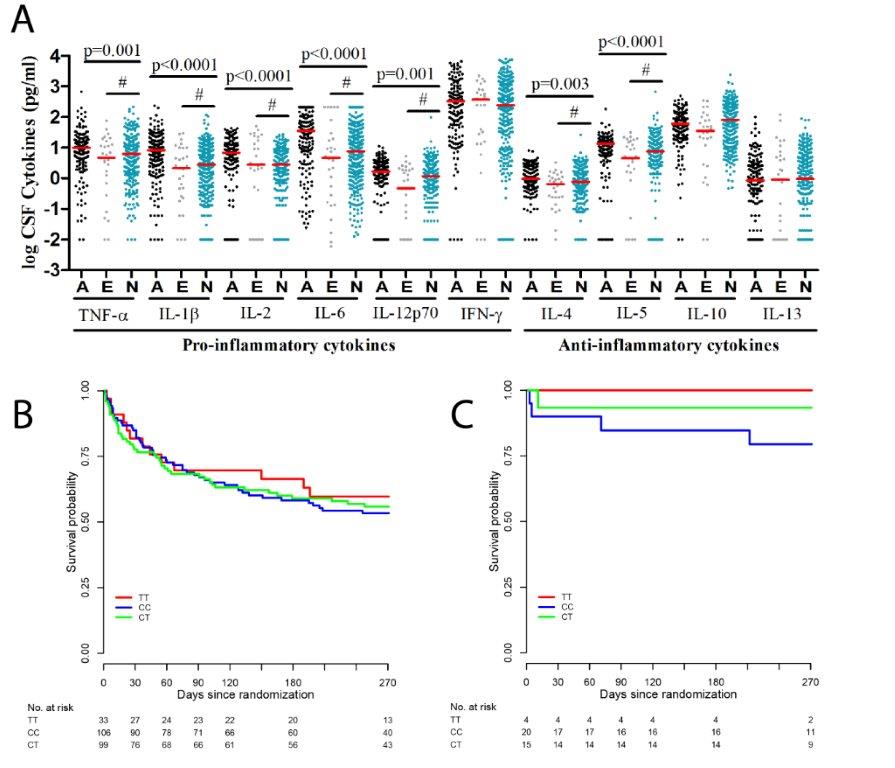

Supplement: Supplementary Figure S2 [file jix050_suppl_Supplementary_Figure_S2.jpeg]
