# Supplementary material for: Leukotriene A4 Hydrolase Genotype and HIV Infection Influence Intracerebral Inflammation and Survival From Tuberculous Meningitis
Source: J Infect Dis. 2017 Apr 17;215(7):1020–8. doi: 10.1093/infdis/jix050 (PMC5426373; doi:10.1093/infdis/jix050)
Supplement: Supplementary Data [file jix050_suppl_Supplementary_Data.docx]

**Supplementary Data**

**Leukotriene A4 hydrolase genotype and HIV infection influence intracerebral inflammation and survival from tuberculous meningitis**

Nguyen TT Thuong^1,2^, Dorothee Heemskerk^1,2^, Trinh TB Tram^1,2^, Le TP Thao^1,2^, Lalita Ramakrishnan^3^, Vu TN Ha^1,2^, Nguyen D Bang^4^, Tran TH Chau^5^, Nguyen H Lan^4^, Maxine Caws^1,6^, Sarah J Dunstan^7^, Nguyen VV Chau^5^, Marcel Wolbers^1,2^, Nguyen TH Mai^5^, Guy E Thwaites^1,2^

**Supplementary Method**

CSF cytokine measurement

A panel of 10 human cytokines was measured in stored CSF by Luminex multiplex bead array technology (Bio-Rad Laboratories, Inc., Hercules, CA, USA). The minimum limits of detection for these cytokines in pg/ml were: IL-1β, 0.01; IL-2, 0.19; IL-4, 0.02; IL-5, 0.18; IL-6, 1.71; IL-10, 0.47; IL-12p70, 0.04; IL-13, 0.02; IFN-γ, 0.17; TNF-α, 0.04. Proportions of cytokines below the level of detection were IL-1β (4.3%), IL-2 (15.9%), IL-4 (2.6%), IL-5 (13.7), IL-6 (0%), IL-10 (0.7%), IL-12p70 (15.9%), IL-13 (18.5%), IFN-γ (4.8%) and TNF-α (0.1%). Cytokine concentrations below the detectable limit were given the value 0.01 pg/ml.

**Supplementary figure legends**

**Figure S1. Kaplan-Meier survival curves stratified by HIV status and BMRC severity grade**

Survival in all patients stratified by HIV status (A) and by BMRC grade (B).

Survival stratified by BMRC grade in HIV-uninfected (C) and HIV-infected patients (D).

Survival in all patients stratified by treatment (E), by treatment in CC genotype (F), in CT genotype (G) and in TT genotype (H).

**Figure S2. (A) CSF cytokine expression levels in HIV-infected patients with advanced disease, CD4 <150 (A), early disease, CD4 ≥150 (E), and HIV-uninfected (N) TBM patients. (B) Kaplan-Meier survival curves stratified by *LTA4H* genotype for HIV-infected with CD4 < 150. (C) as (B) for CD4 ≥ 150.**

(A) Statistical comparisons between HIV-uninfected (n=310) and either HIV-infected with CD4 <150 (n=173) or CD4 **≥**150 (n=28) were made using a linear trend test corrected for multiple testing across the 10 cytokines; #, p>0.05. (B) CD4 <150 cases comprise 106CC, 100CT, 33TT. (C) CD4 ≥ 150 comprise 20CC, 15CT, 4TT.

**Supplementary tables**

**Table S1. Baseline clinical characteristics of all patients and stratified by HIV status**

|  | **All patients (N = 764)** | | **HIV-uninfected (N = 439)** | | **HIV-infected (N = 325)** | | Comparison  p-value |
| --- | --- | --- | --- | --- | --- | --- | --- |
| Characteristic | n | Summary statistic | n | Summary statistic | n | Summary statistic |  |
| Male sex – no. (%) | 764 | 526 (68.8%) | 439 | 265 (59.7%) | 325 | 265 (81.2%) | < 0.0001 |
| Age (years) – median (IQR) | 764 | 35 (29,46) | 439 | 41 (28,55) | 325 | 33 (30,38) | < 0.0001 |
| Weight (kg) – median (IQR) | 764 | 48.0 (44.0, 54.0) | 439 | 49.0 (45.0, 55.0) | 325 | 47.4 (43.0, 52.0) | 0.013 |
| Previous tuberculosis – no. (%) † | 764 | 136 (17.8%) | 439 | 46 (10.9%) | 325 | 90 (27.7%) | < 0.0001 |
| Duration of illness (days) – median (IQR) | 761 | 15 (10,30) | 438 | 15 (10,26) | 323 | 15 (9,30) | 0.79 |
| Treatment arm | 764 |  | 439 |  | 325 |  | 0.94 |
| - Standard |  | 386 (50.5%) |  | 221 (50.3%) |  | 165 (50.7%) |  |
| - Intensified |  | 378 (49.5%) |  | 218 (49.7%) |  | 160 (49.2%) |  |
| *ART at enrolment | - |  | - |  | 325 |  | - |
| - not on ART at enrolment |  | - |  | - |  | 212 (65.2%) |  |
| - on ART at enrolment |  | - |  | - |  | 113 (34.8%) |  |
| **BMRC grade no. (%) | 764 |  | 439 |  | 325 |  | 0.05 |
| - Grade 1 |  | 300 (39.3%) |  | 163 (37.1%) |  | 137 (42.1%) |  |
| - Grade 2 |  | 330 (43.2%) |  | 206 (46.9%) |  | 124 (38.1%) |  |
| - Grade 3 |  | 134 (17.5%) |  | 70 (15.9%) |  | 64 (19.7%) |  |
| Diagnostic category – no. (%) †† | 764 |  | 439 |  | 325 |  | < 0.0001 |
| - definite TBM |  | 396 (51.8%) |  | 188 (42.8%) |  | 208 (64.0%) |  |
| - probable TBM |  | 204 (26.7%) |  | 133 (30.3%) |  | 71 (21.8%) |  |
| - possible TBM |  | 164 (21.5%) |  | 118 (26.9%) |  | 46 (14.1%) |  |
| Resistance category – no. (%) | 764 |  | 439 |  | 325 |  | < 0.0001 |
| - no or other resistance |  | 213 (27.9%) |  | 91 (20.7%) |  | 122 (37.5%) |  |
| - INH resistant ‡ |  | 85 (11.13%) |  | 38 (8.7%) |  | 47 (14.5%) |  |
| - RIF resistant/MDR § |  | 15 (2.00%) |  | 3 (0.7%) |  | 12 (3.7%) |  |
| - unknown resistance ¶ |  | 451 (59.0%) |  | 307 (69.9%) |  | 144 (44.3%) |  |
| Blood - median (IQR) |  |  |  |  |  |  |  |
| -Sodium (mmol/l) | 696 | 128 (123,132) | 401 | 129 (124,133) | 295 | 126 (121,131) | < 0.0001 |
| -Leucocytes total (x 10^6^ cells/ml) | 720 | 9.0 (6.6, 12.1) | 418 | 10.4 (8.2, 13.3) | 302 | 7.1 (5.4, 9.9) | < 0.0001 |
| -Neutrophils (%) | 720 | 70.0 (72.0,86.1) | 418 | 80.9 (72.8,87.0) | 302 | 78.0 (71.1,84.9) | 0.01 |
| -Lymphocytes (%)  -CD4 cell count (x 10^3^ cells/ml) | 720  - | 11.7 (7.2,16.8)  - | 418  - | 11.2 (6.9,16.5)  - | 302  278 | 12.2 (8.0,17.1)  38 (15,101) | 0.22  - |
| Cerebrospinal fluid - median (IQR) |  |  |  |  |  |  |  |
| -Leucocytes total (x 10^3^ cells/ml) | 738 | 115 (35,278) | 425 | 112 (38,256) | 313 | 124 (28,340) | 0.36 |
| -Neutrophils (%) | 704 | 10 (0,32) | 412 | 5 (0,20) | 292 | 17 (0,43) | < 0.0001 |
| -Lymphocytes (%) | 704 | 90 (69,100) | 412 | 95 (80,100) | 292 | 83 (57,100) | < 0.0001 |
| -Protein (g/l) | 715 | 1.20 (0.60,1.93) | 412 | 1.04 (0.53,1.72) | 303 | 1.40 (0.77,2.20) | < 0.0001 |
| -Glucose (mmol/l) | 716 | 1.90 (1.27,2.70) | 414 | 2.04 (1.30,2.90) | 302 | 1.71 (1.15,2.49) | 0.0005 |
| -Lactate (mmol/l) | 663 | 4.80 (3.50,6.47) | 385 | 4.80 (3.60,6.40) | 278 | 4.88 (3.32,6.58) | 0.92 |
| LTA4H genotype– no. (%) | 764 |  | 439 |  | 325 |  | 0.06 |
| - TT |  | 89 (11.6%) |  | 43 (9.8%) |  | 46 (14.1%) |  |
| - CT |  | 345 (45.2%) |  | 212 (48.3%) |  | 133 (40.9%) |  |
| - CC |  | 330 (43.2%) |  | 184 (42.0%) |  | 146 (44.9%) |  |

All summary statistics are absolute counts (%) for categorical variables and median (inter-quartile range = IQR) for continuous data. n refers to the number of patients with non-missing data for the corresponding variable.

p values are descriptive only and based on Fisher's exact test for categorical data and Wilcoxon's test for continuous data.

† Patients who reported having treatment for a previous episode of disease.

* ART; anti-retroviral therapy

** BMRC denotes modified British Medical Research Council criteria. Grade1 indicates a Glasgow coma score of 15 with no neurologic signs (baseline), grade 2 a score of 11 to 14 (or 15 with focal neurologic signs), and grade 3 a score of 10 or less.

††Diagnostic categories were assigned according to the consensus case definition.[^1^](#_ENREF_1) Patients with an unlikely diagnosis of tuberculous meningitis had a score of <6. Confirmed other diagnosis was only made based on microbiological evidence.

‡ Resistance to isoniazid (INH), with or without other resistance, but not rifampicin resistance

§ MDR; multi-drug resistance to at least rifampicin and isoniazid, or isolated rifampicin resistance from culture results.

¶ Patients for whom no drug resistance results were available.

Reference

1. Marais S, Thwaites G, Schoeman JF, Torok ME, Misra UK, Prasad K, et al. Tuberculous meningitis: a uniform case definition for use in clinical research. The Lancet Infectious diseases. 2010; **10**(11): 803-12.

**Table S2. Characteristics of baseline variables for three *LTA4H* genotypes in all patients**

| Characteristic | n | **Summary statistic** | **n** | **Summary statistic** | **n** | **Summary statistic** | **Comparison** |
| --- | --- | --- | --- | --- | --- | --- | --- |
|  |  | **TT (N = 89)** |  | **CT (N = 345)** |  | **CC (N = 330)** | **p-value** |
| Male sex – no. (%) | 89 | 65 (73.0%) | 345 | 241 (69.9%) | 330 | 220 (66.7%) | 0.46 |
| Age (years) – median (IQR) | 89 | 35 (31,45) | 345 | 36 (29,49) | 330 | 35 (29,44) | 0.31 |
| Weight (kg) – median (IQR) | 89 | 50 (45,55) | 345 | 49 (44,54) | 330 | 48(44,53) | 0.43 |
| Previous tuberculosis – no. (%) | 89 | 11 (12.4%) | 345 | 65(18.8%) | 330 | 60 (18.2%) | 0.37 |
| Duration of illness (days) –  median (IQR) | 88 | 15 (10,22) | 345 | 15 (10,30) | 328 | 15 (9,30) | 0.47 |
| Treatment arm | 89 |  | 345 |  | 330 |  | 0.93 |
| - Standard |  | 44 (49.4%) |  | 177 (51.3%) |  | 165 (50.0%) |  |
| - Intensified |  | 45 (50.6%) |  | 168 (48.7%) |  | 165(50.0%) |  |
| BMRC grade no. (%) | 89 |  | 345 |  | 330 |  | 0.63 |
| - Grade 1 |  | 40 (44.9%) |  | 137 (39.7%) |  | 123 (37.3%) |  |
| - Grade 2 |  | 35 (39.3%) |  | 152 (44.1%) |  | 143 (43.3%) |  |
| - Grade 3 |  | 14 (15.7%) |  | 56 (16.2%) |  | 64 (19.4%) |  |
| Diagnostic category – no. (%) | 89 |  | 345 |  | 330 |  | 0.68 |
| - definite TBM |  | 49 (55.1%) |  | 170 (49.3%) |  | 177 (53.6%) |  |
| - probable TBM |  | 20 (22.5%) |  | 97 (28.1%) |  | 87 (26.4%) |  |
| - possible TBM |  | 20 (22.5%) |  | 78 (22.6%) |  | 66 (20.0%) |  |
| Resistance category – no. (%) | 89 |  | 345 |  | 330 |  | 0.87 |
| - no or other resistance |  | 30 (33.7%) |  | 91 (26.4%) |  | 92 (27.9%) |  |
| - INH resistant |  | 9 (10.1%) |  | 41 (11.9%) |  | 35 (10.6%) |  |
| - RIF resistant/MDR |  | 2 (2.3%) |  | 6 (1.7%) |  | 7 (2.1%) |  |
| - unknown resistance |  | 48 (53.9%) |  | 207 (60%) |  | 196 (59.4%) |  |
| HIV | 89 |  | 345 |  | 330 |  | 0.06 |
| - negative |  | 43 (48.3%) |  | 212 (61.5%) |  | 189 (55.8%) |  |
| - positive |  | 46 (51.7%) |  | 134 (38.5%) |  | 146 (44.2%) |  |
| Blood - median (IQR) |  |  |  |  |  |  |  |
| -Sodium (mmol/l) | 79 | 127 (124,131) | 318 | 128 (123,132) | 299 | 128 (123,133) | 0.45 |
| -Leucocytes total (x 10^6^ cells/ml) | 82 | 8.1 (5.6,10.3) | 329 | 8.9 (6.7,12.1) | 309 | 9.6 (6.8,12.6) | 0.01 |
| -Neutrophils (%) | 82 | 78.5 (68.8,84.7) | 329 | 80.4 (72.6,86.3) | 309 | 80.1 (72.2,86.4) | 0.20 |
| -Lymphocytes (%) | 82 | 12.9 (8.6,17.5) | 329 | 11.6 (6.9,16.4) | 309 | 11.2 (7.2,17.0) | 0.19 |
| CSF - median (IQR) |  |  |  |  |  |  |  |
| -Leucocytes total (x 10^3^ cells/ml) | 86 | 118 (27,294) | 331 | 128 (40,293) | 321 | 102 (32,258) | 0.46 |
| -Neutrophils (%) | 80 | 10 (0,38) | 317 | 9.0 (0,28) | 307 | 10 (0,33) | 0.44 |
| -Lymphocytes (%) | 80 | 90 (63,100) | 317 | 90 (72,100) | 307 | 90 (67,100) | 0.54 |
| -Protein (g/l) | 81 | 1.30 (0.57,2.09) | 327 | 1.10 (0.60,1.90) | 307 | 1.23 (0.70,1.92) | 0.63 |
| -Glucose (mmol/l) | 81 | 1.90 (1.28,2.90) | 328 | 1.90 (1.30,2.67) | 307 | 1.90 (1.20,2.70) | 0.99 |
| -Lactate (mmol/l) | 75 | 5.06 (3.30,6.80) | 302 | 4.68 (3.48,6.37) | 286 | 4.90 (3.60,6.60) | 0.62 |

**Table S3. Characteristics of baseline variables for three *LTA4H* genotypes in HIV-uninfected patients**

| **Characteristic** | **n** | **Summary statistic** | **n** | **Summary statistic** | **n** | **Summary statistic** | **Comparison** |
| --- | --- | --- | --- | --- | --- | --- | --- |
|  |  | **TT (N=43)** |  | **CT (N=212)** |  | **CC (N=184)** | **p-value** |
| Male sex – no. (%) | 43 | 25 (58.1%) | 212 | 135 (63.7%) | 184 | 102 (55.4%) | 0.24 |
| Age (years) – median (IQR) | 43 | 45 (34,54) | 212 | 43 (29,58) | 184 | 38 (26,51) | 0.11 |
| Weight (kg) – median (IQR) | 43 | 50 (45,56) | 212 | 50 (45,55) | 184 | 48 (44,55) | 0.55 |
| Previous tuberculosis – no. (%) | 43 | 2 (4.7%) | 212 | 24 (11.3%) | 184 | 20 (10.9%) | 0.47 |
| Duration of illness (days) –  median (IQR) | 43 | 14 (10,20) | 212 | 15 (10,29) | 183 | 15 (9,26) | 0.28 |
| Treatment arm | 43 |  | 212 |  | 184 |  | 0.85 |
| - Standard |  | 23 (53.5%) |  | 104 (49.1%) |  | 94 (51.1%) |  |
| - Intensified |  | 20 (46.5%) |  | 108 (50.9%) |  | 90 (48.9%) |  |
| BMRC grade no. (%) | 43 |  | 212 |  | 184 |  | 0.74 |
| - Grade 1 |  | 18 (41.9%) |  | 81 (38.2%) |  | 64 (34.8%) |  |
| - Grade 2 |  | 20 (46.5%) |  | 100 (47.2%) |  | 86 (46.7%) |  |
| - Grade 3 |  | 5 (11.6%) |  | 31 (14.6%) |  | 34 (18.5%) |  |
| Diagnostic category – no. (%) | 43 |  | 212 |  | 184 |  | 0.56 |
| - definite TBM |  | 20 (46.5%) |  | 82 (38.7%) |  | 86 (46.7%) |  |
| - probable TBM |  | 13 (30.2%) |  | 69 (32.5%) |  | 51 (27.7%) |  |
| - possible TBM |  | 10 (23.3%) |  | 61 (28.8%) |  | 47 (25.5%) |  |
| Resistance category – no. (%) | 43 |  | 212 |  | 184 |  | 0.92 |
| - no or other resistance |  | 10 (23.3%) |  | 45 (21.2%) |  | 36 (19.6%) |  |
| - INH resistant |  | 2 (4.7%) |  | 18 (8.5%) |  | 18 (9.8%) |  |
| - RIF resistant/MDR |  | 0 (0%) |  | 1 (0.5%) |  | 2 (1.1%) |  |
| - unknown resistance |  | 31 (72.1%) |  | 148 (70.1%) |  | 128 (69.6%) |  |
| Blood - median (IQR) |  |  |  |  |  |  |  |
| -Sodium (mmol/l) | 40 | 128 (124,132) | 193 | 129 (124,133) | 168 | 130 (125,134) | 0.48 |
| -Leucocytes total (x 10^6^ cell/µl) | 41 | 9.1 (7.8,11.3) | 202 | 10.2 (8.2,13.0) | 175 | 10.7 (8.3,14.0) | 0.04 |
| -Neutrophils (%) | 41 | 79.8 (68.4,84.5) | 202 | 81.2 (72.9,87.2) | 175 | 81.1 (73.9,87.5) | 0.31 |
| -Lymphocytes (%) | 41 | 12.9 (8.3,17.8) | 202 | 11.2 (6.9,16.2) | 175 | 11.0 (6.7,16.6) | 0.38 |
| CSF- median (IQR) |  |  |  |  |  |  |  |
| -Leucocytes total (x 10^3^ cell/ml) | 43 | 117 (38,269) | 204 | 114 (40,266) | 178 | 104 (36,239) | 0.81 |
| -Neutrophils (%) | 42 | 8 (0,18) | 197 | 5 (0,20) | 173 | 5 (0,20) | 0.98 |
| -Lymphocytes (%) | 42 | 92 (83,100) | 197 | 95 (80,100) | 173 | 95 (80,100) | 0.98 |
| -Protein (g/l) | 41 | 0.90 (0.42,1.70) | 199 | 1.03 (0.54,1.82) | 172 | 1.10 (0.58,1.61) | 0.63 |
| -Glucose (mmol/l) | 41 | 2.10 (1.36,3.10) | 201 | 2.00 (1.30,2.80) | 172 | 2.08 (1.38,2.90) | 0.82 |
| -Lactate (mmol/l) | 40 | 5.25 (3.98,7.05) | 183 | 4.70 (3.40,6.39) | 162 | 4.85 (3.70,6.20) | 0.57 |

**Table S4. Characteristics of baseline variables for three *LTA4H* genotypes in HIV-infected patients**

| **Characteristic** | **n** | **Summary statistic** | **n** | **Summary statistic** | **n** | **Summary statistic** | **Comparison** |
| --- | --- | --- | --- | --- | --- | --- | --- |
|  |  | **TT (N=46)** |  | **CT (N=133)** |  | **CC (N=146)** | **p-value** |
| Male sex – no. (%) | 46 | 40 (87.0%) | 133 | 106 (79.7%) | 146 | 118 (80·8%). | 0.57 |
| Age (years) – median (IQR) | 46 | 33 (30,38) | 133 | 33 (29,37) | 146 | 34 (30,38) | 0.54 |
| Weight (kg) – median (IQR) | 46 | 48 (43,55) | 133 | 48 (42,54) | 146 | 47 (43,50) | 0.73 |
| Previous tuberculosis – no. (%) | 46 | 9 (19.6%) | 133 | 41 (30.8%) | 146 | 40 (27.4%) | 0.35 |
| Duration of illness (days) –  median (IQR) | 45 | 15 (9,30) | 133 | 15 (9,30) | 145 | 15 (9,30) | 0.97 |
| Treatment arm | 46 |  | 133 |  | 146 |  | 0·43 |
| - Standard |  | 21 (45.7%) |  | 73 (54.9%) |  | 71 (48.6%) |  |
| - Intensified |  | 25 (54.4%) |  | 60 (45.1%) |  | 75 (51.4%) |  |
| ART at enrolment | 46 |  | 133 |  | 146 |  | 0.94 |
| - not on ART at enrolment |  | 29 (63.0%) |  | 87 (65.4%) |  | 96(65.8%) |  |
| - on ART at enrolment |  | 17 (37.0%) |  | 46 (34.6%) |  | 50(34.3%) |  |
| BMRC grade no. (%) | 46 |  | 133 |  | 146 |  | 0.91 |
| - Grade 1 |  | 22 (47.8%) |  | 56 (42.1%) |  | 59(40.4%) |  |
| - Grade 2 |  | 15 (32.6%) |  | 52 (39.1%) |  | 57(39.0%) |  |
| - Grade 3 |  | 9 (19.6%) |  | 25 (18.8%) |  | 30(20.6%) |  |
| Diagnostic category – no. (%) | 46 |  | 133 |  | 146 |  | 0.45 |
| - definite TBM |  | 29 (63.0%) |  | 88 (66.2%) |  | 91 (62.3%) |  |
| - probable TBM |  | 7 (15.2%) |  | 28 (21.0%) |  | 36 (24.7%) |  |
| - possible TBM |  | 10 (21.7%) |  | 17 (12.8%) |  | 19 (13.0%) |  |
| Resistance category – no. (%) | 46 |  | 133 |  | 146 |  | 0.76 |
| - no or other resistance |  | 20 (43.5%) |  | 46 (34.6%) |  | 56 (38.4%) |  |
| - INH resistant |  | 7 (15.2%) |  | 23 (17.3%) |  | 17 (11.6%) |  |
| - RIF resistant/MDR |  | 2 (4.4%) |  | 5 (3.8%) |  | 5 (3.4%) |  |
| - unknown resistance |  | 17 (37.0%) |  | 59 (44.4%) |  | 68 (46.6%) |  |
| Blood - median (IQR) |  |  |  |  |  |  |  |
| -Sodium (mmol/l) | 39 | 126 (124,130) | 125 | 126 (121,131) | 131 | 126(122,132) | 0.51 |
| -CD4 cell count | 37 | 31.0 (14.0,86.0) | 114 | 39.5 (13.2,82.2) | 126 | 43.0 (17.5,110.8) | 0.36 |
| -Leucocytes total (x 10^6^ cell/ml) | 41 | 6.9(4.4,9.1) | 127 | 7.0 (5.6,9.2) | 134 | 7.4 (5.5,10.2) | 0.32 |
| -Neutrophils (%) | 41 | 76.7 (68.9,84.8) | 127 | 78.1 (72.5,84.6) | 134 | 78.1(70.1,85.3) | 0.62 |
| -Lymphocytes (%) | 41 | 12.9 (10.0,17.3) | 127 | 12.3 (7.2,16.7) | 134 | 12.0 (8.3,17.1) | 0.55 |
| CSF- median (IQR) |  |  |  |  |  |  |  |
| -Leucocytes total (x 10^3^ cell/ml) | 43 | 120 (25,440) | 127 | 144 (38,338) | 143 | 102 (26,283) | 0.47 |
| -Neutrophils (%) | 38 | 27 (4,52) | 120 | 10 (0,42) | 134 | 20 (0,43) | 0.26 |
| -Lymphocytes (%) | 38 | 75 (47,98) | 120 | 90 (59,100) | 134 | 80 (58,100) | 0.39 |
| -Protein (g/l) | 40 | 1.42 (0.88,2.21) | 128 | 1.25 (0.67,1.98) | 135 | 1.58 (0.82,2.44) | 0.09 |
| -Glucose (mmol/l) | 40 | 1.75 (1.19,2.46) | 127 | 1.69 (1.30,2.45) | 135 | 1.78 (1.10,2.50) | 0.97 |
| -Lactate (mmol/l) | 35 | 4.70 (2.42,6.10) | 119 | 4.60 (3.50,6.31) | 124 | 5.00 (3.48,7.12) | 0.41 |
